# East Tennessee Dental Association

**Published:** 1874-12

**Authors:** S. B. Cooke

**Affiliations:** Secretary.


### ARTICLE III.

### *East Tennesee Dental Association.*

The Eighth Annual Meeting of the East Tennesee Dental Association met in Knoxville, September 16th, 1874.

President Dr. M. F. Fowler in the chair.

Dr. Fowler read an essay on Pivot teeth. Dr. W. H.

Cooke one on Dental Neuralgia, and Dr. Protho one on Diagnosis. The essays were all ably discussed.

The following Resolution was unanimously adopted:

WHEREAS, Dr. John Foucke, of Knoxville, has been untiring in his efforts for the advancement of the Dental Profession, as well as each individual member of this Society ever since its organization; and, WHEREAS, we are indebted to him for valuable instructions, especially for his invention of the proper manner of preparing soft gold for building up teeth; Therefore, Resolved that the thanks of this Society are hereby tendered him, and we will ever hold him in grateful remembrance.

A Resolution was adopted requiring all the junior members to be examined on Human Anatomy, at the next annual meeting.

The following were elected officers for the ensuing year:

Dr. S. M. Protho, President; Dr. R. A. B. Myers, Vice President; Dr. S. B. Cooke, Recording Secretary and Treasurer; Dr. M. M. Harris, Corresponding Secretary.

On motion the Association adjourned.

Clinics were held daily by Drs. W. H. Cooke, R. A. B. Myers, and S. B. Cooke.

S. B. COOKE, *Secretary.*

